# Antemortem Diagnosis of New York Human Rabies Case and Review of U.S. Cases

**Published:** 2006-12

**Authors:** Vince V. Soun, Millicent Eidson, Barbara J. Wallace, Peter D. Drabkin, Ginelle Jones, Richard Leach, Kathy Cantiello, Charles V. Trimarchi, Jiang Qian

**Affiliations:** 1 *Bureau of Communicable Disease Control, New York State Department of Health, Albany, New York, USA;*; 2 *Capital District Regional Office, New York State Department of Health, Troy, New York, USA;*; 3 *Warren County Public Health Services, Lake George, New York, USA;*; 4 *Glens Falls Hospital, Glens Falls, New York, USA;*; 5 *Laboratory of Zoonotic Disease and Clinical Virology, Wadsworth Center, New York State Department of Health, Guilderland, New York, USA;*; 6 *Department of Pathology and Laboratory Medicine, Albany Medical Center, Albany, New York, USA*

**Keywords:** dog diseases, fluorescent antibody technique, inclusion bodies, rabies, reverse transcriptase polymerase chain reaction, virus disease

## Abstract

To help elucidate rabies disease patterns and control issues, a full assessment of a human case of dog-variant rabies was undertaken. In 2000, a 54-year-old man presented to a New York hospital with lower back discomfort four days after arrival from Africa. Rabies was first suspected 8 days after hospitalization based on clinical signs, specimens were collected on the same day, and rabies infection was confirmed the following day (fluorescence antibody testing on nuchal skin biopsy specimen). By the 12^th^ day after illness onset, he was unresponsive, and life support was removed on day 15. Subsequently, an African dog variant was confirmed by nucleic acid sequence analysis of rabies viral RNA extracted and amplified from the patient’s saliva. Management of human concerns about exposure to the patient kept the number of persons receiving postexposure prophylaxis to 26. With less than half of the U.S. human rabies cases being diagnosed antemortem, this case emphasizes the need to routinely include rabies in the differential diagnosis of any unexplained encephalitis to ensure early confirmation and triage of human contacts to reduce associated healthcare costs.

## INTRODUCTION

From the 1980-2004, the U.S. has reported 56 cases of human rabies (1). Rabies is a preventable viral disease that causes an acute, progressive, fatal inflammation of the central nervous system (CNS) in mammals, including humans if appropriate preventive treatment is not received prior to or soon after exposure. Part of the genus *Lyssavirus* in the *Rhabdoviridae* family, the prototype virus is primarily transmitted by the bite of a rabid animal through introduction into the wound of infectious virus contained in the animal’s saliva. Rabies virus is maintained in nature in the form of numerous variants that cycle in a predominant vector species in geographically and temporally defined outbreaks. The variants and the rabies cycles are named after the primary vectors, which in North America currently include bats, skunks, raccoons, and foxes. Each variant is capable of infecting other mammal species, including humans.

Early signs and symptoms of the disease in infected humans are nonspecific, ranging from fever and headache to general malaise. However, as the disease progresses, neurological signs such as insomnia, anxiety, confusion, slight or partial paralysis, excitation, delirium, and hydrophobia may ensue (2, 3). For many reasons, human rabies cases may be difficult to identify and work up antemortem, including the lack of specificity of early signs, the need to identify appropriate laboratory tests, issues with their sensitivity early in the clinical course, and the high case fatality rate leading to poor patient prognosis and high concern among human contacts (2, 3).

A full assessment of an imported human case of dog-variant rabies in New York State (NYS) is undertaken to help elucidate clinical aspects of the disease to assist in antemortem diagnosis, and to provide methods for addressing human-to-human exposures.

## PATIENT AND METHODS

Supporting documents for the case were obtained from the medical record and from case investigators. The initial antemortem laboratory diagnosis was made by immunofluorescence microscopic examination performed on cryostat-cut frozen sections of full thickness skin punch biopsy taken in the nuchal area, performed at the NYS Department of Health (NYSDOH) Wadsworth Center Rabies Laboratory. Microscopic examination of tissues stained by the direct immunofluorescence method employing FITC-labeled rabies virus nucleocapsid protein-specific antibodies is the gold standard for detection of rabies virus in postmortem examination of animal or human CNS specimens, and has proven to be sensitive and specific when applied to skin biopsy or corneal impression specimens for antemortem diagnosis of human rabies, particularly further into the clinical period of rabies infection. With results available in as short as two hours from receipt of the specimens at the laboratory, this method is extremely valuable in the rapid identification of rabies infection.

The immunofluorescence method employed has been described elsewhere (4). The molecular characterization was performed by reverse transcriptase polymerase chain reaction (RT-PCR) and sequence analysis of the N gene in RNA extracted from the patient’s saliva as described elsewhere (5). Detection of rabies virus RNA extracted from clinical samples such as saliva, that are not suitable for immunofluorescence microscopy, has proven to be the most sensitive method for the antemortem diagnosis of rabies in humans (6). Subsequent analysis of the nucleic acid sequences of the specific products of the amplification process permits comparison with published sequences associated with rabies virus variants identified with specific cycles of rabies virus maintained in characteristic vector species populations and geographic locations. The University of Wisconsin Genetics Computer Group (GCG) software package computer analysis was used to perform pairwise comparisons that permitted estimates of genetic identity with published sequences for dog rabies isolates of African origin (7). Postmortem confirmation was done by immunofluorescence microscopy on brain tissue, histologic examination of hematoxylin and eosin stained sections of paraffin-embedded cerebellum, cerebral cortex, brainstem and spinal cord, and electron microscopy performed at 40,000 times magnification (8).

For a comparison with the NY case, 46 U.S. human rabies cases reported from 1989-1994 were reviewed.

## RESULTS

**Figure 1:**
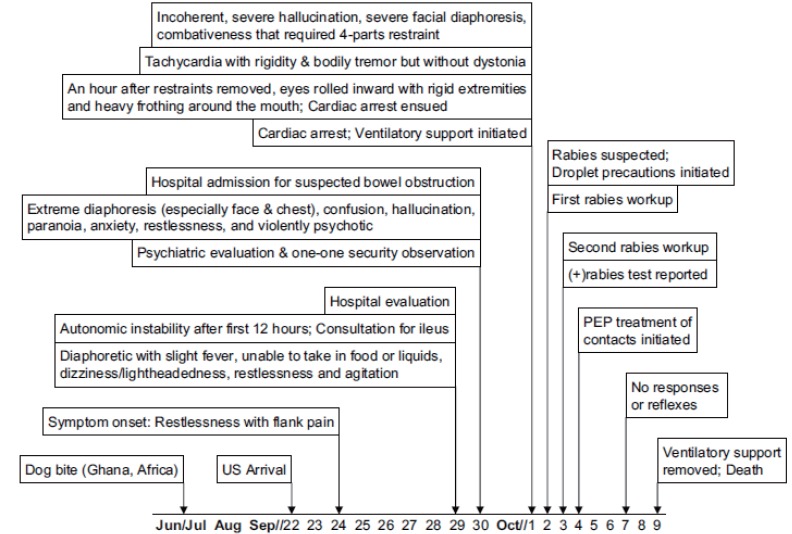
Timeline of the year 2000 NY imported dog-variant rabies case in relation to onset of symptoms.

In late June or early July 2000, one of six unvaccinated puppies bit its 54-year-old male owner on his right thumb and right leg at his home in Ghana before his departure for an exchange professorship to upstate NY in September (Fig. 1). The first possible indication of infection occurred on September 24 (day 0), two days after arrival in NYS when he began to feel restless and a need to sleep on the couch that night. He awoke the next morning with flank pain that persisted intermittently until September 27 (day 3) when it began to intensify along with abdominal pain. The pain increased with restlessness and agitation without evidence of frantic or aggressive behavior. With intensification of abdominal pain, an emergency call was placed at 1a.m. on September 29 (day 5). The patient was evaluated in the emergency room (ER) during the first 24 hours and was formally admitted in the early morning of September 30 (day 6) for suspected bowel obstruction.

At the ER, the initial examination was normal except for mild distress associated with diaphoresis and anxiety (Table 1). The patient denied any prior fevers, cough, recent chills or illness. Furthermore, he had not experienced any lightheadedness, palpitations, chest pain or weakness. Physical examination revealed he had soft tenderness in the right abdominal area and no evidence of a pulsatile mass. The patient’s peripheral white blood cell (WBC) count was in the normal range but a relative monocytosis and lymphopenia were noted (Table 2). Blood glucose, CO_2_, mean corpuscular hemoglobin (MCH), and aspartate transaminase (AST) were elevated. Urinalysis revealed traces of protein but the urine was negative for WBCs, red blood cell (RBCs), and bacteria (Table 3). The specific gravity (s.g.) was low. Three attempts were made to obtain a cerebral spinal fluid (CSF) specimen, but these were unsuccessful. Computerized tomography (CT) performed at 3:10 a.m. on September 29 revealed a few gas-filled distended small bowel loops projected over the mid abdomen (later interpreted as aerophagia). Air fluid levels were present on upright and decubitus views in both the large and small bowel. No pneumoperitoneum was identified. Repeat urinalysis demonstrated a low s.g. and an elevated pH and protein level (Table 3). No nitrite, leukocytes, epithelial cells, WBCs, RBCs, bacteria, mucus, or casts were detected.

**Table 1:** Vital signs for the 2000 NY human rabies case (abnormal values are bolded), September-October 2000

	9/29^a^	9/29^b^	9/30^c^	10/1^d^	10/2^e^	10/3^f^	10/4^g^	Normal Range

Respiratory	18	20	26	---	---	---	---	12-20 rpm
Temperature	---	37.7°C	38.8°C	---	---	---	36.0°C	35.8°C-37.3°C
Pulse	88	96	108	102	127, 81	97, 102	90	60-100 bpm
Pupils	Normal	---	---	---	---	---	---	Normal
Blood Pressure	128/76	175/95	190/113	---	---	---	110/55	120/80
Oxygen Saturation	---	96	99	100	97, 96	95, 98	98	100%

---, data is not available or is missing;

a Vital signs taken in ambulance at 1:04 a.m;

b Vital signs taken in the hospital emergency room around 1:30 p.m. (12 hours post-arrival);

c Vital signs taken after hospital admission at 7:45 a.m;

d Pulse and oxygen saturation were taken at 10:00 a.m.; From October 1-9, patient was on mechanical ventilation;

e Pulse and oxygen saturation were taken at 1:00 a.m. and 3:00 a.m., respectively;

f Pulse and oxygen saturation were taken at 1:10 p.m. and 3:00 p.m., respectively;

g Values reflect samples collected between 5:30 a.m. and 6:10 a.m.

**Table 2:** Blood chemistry findings for the 2000 NY human rabies case (abnormal values are bolded; H=high, L=low), September-October 2000

	9/29^a^	9/30^b^	10/1^c^	10/2^d^	10/3^e^	10/4^f^	Normal Range^g^

Blood glucose	134H	167H	137H, 303H	122H	125H	143H	70-110 mg/dL
Blood urea	13	5	25H, 25H	29H	23	19	7-24 mg/dL
Creatinine	1.3	1.2	1.3, 1.5H	1.7H	1.5H	1.1	0.6-1.3 mg/dL
Sodium	141	138	146H, 145	148H	150H	151H	136-145 mmol/L
Potassium	3.4L	3.9	4.3, 3.9	4	4.1	3.7	3.5-5.1 mmol/L
Chloride	102	99L	109, 115H	108	110H	113H	100-109 mmol/L
Calcium	9	9.5	10.2, 7.8L	9.2	9.2	9.1	8.5-10.2 mg/dL
CO_2_	33H	26	27, 20	33H	36H	32	18-32 mmol/L
WBCs (× 10^3^)	7.8	---	9.4, 5.5	12.3H	10.9H	7.9	4.8-10.8 × 10^3^/uL
Neutrophils	68.1	---	70.5	---	---	---	44.0-74.0%
Lymphocytes	19.7L	---	25, 17.1L	12L	---	15L	24.0-44.0%
Basophils	0.8	---	0.8	---	---	---	0.0-2.0%
Monocytes	11.3H	---	11H,11.4H	6.0	---	13H	0.0-10.0%
Eosinophils	0.1	---	0.2	---	---	---	0.0-5.0%
RBCs (× 10^6^)	4.79	5.04	5.34, 3.91L	4.74	4.28L	3.98L	4.70-6.10 × 10^6^/uL
Hemoglobin	15.5	16.5	17.3, 12.6L	15.4	13.9L	13.1L	14.0-18.0g/dL
Hematocrit	44.7	46.8	49.3, 36.2L	44	39.9L	37.5L	42.0-52.0%
Platelets	161	179	197, 158	200	173	157	130-440 × 10^3^/uL
MCV	93.1	92.7	92.3, 92.6	92.8	93.2	94	81.0-99.0 fL
MCH	32.3H	32.7H	32.3H, 32.3H	32.4H	32.5H	32.9H	27.0-31.0 pg
AST	59H	---	180H, 115H	107H	---	---	12-32 U/L
Arterial Blood Gas (ABG)*	---	---	See comment below*	---	---	---	See comment below*

* Analysis of patient on ventilatory support: pH7.39 (normal: 7.35-7.45)^g^, pO^2^ 123 on oxygen, pCO^2^ 42 (normal: 36-44 mmHg)^g^, base excess 0 (normal -2 to +2 mEq/L)^g^.

---, data is not available or is missing; MCV = Mean Corpuscular Volume; MCH, Mean Corpuscular Hemoglobin; AST, Aspartate Transaminase;

a Samples taken in the hospital emergency room at 2:56 a.m.; samples for PLT and AST were taken at 9:56 a.m;

b Samples taken after hospital admission at 6:00 a.m;

c Samples taken at 7:59 a.m. and 10:47 a.m., respectively;

d Samples taken at 5:25 a.m;

e Samples taken at 5:10 a.m;

f Samples taken between 5:30 a.m. and 6:10 a.m;

g Reference values were based on the clinical laboratory of Glens Falls Hospital, Warren County, NY.

**Table 3:** Laboratory findings for the 2000 NY human rabies case (abnormal values are bolded; H=high, L=low), September-October 2000

	9/29^a^	9/30^b^	10/1^c^	10/2	10/3	10/4	Normal Range

pH	6.5, 8.5H	---	---	---	---	---	4.5-7.8^d^
s.g.	1.00L, 1.012L	---	---	---	---	---	1.015-1.030^e^
Protein	tr, 30	---	100	---	---	---	24-133 mg/24h^d^*
Glucose	---	---	>1000H	---	---	---	1-15 mg/100 mL^d^
Sed rate	---	---	28H	---	---	---	0 - 15 mm/hr^e^

---, data is not available or is missing; s.g., specific gravity; tr, trace;

* soluble protein in urine;

a Samples were taken in the hospital emergency room at 2:56 a.m. and 3:52 a.m., respectively;

b Samples were taken after hospital admission;

c Samples were taken at 9:00 a.m.;

d Reference #50;

e Reference values were based on the clinical laboratory of Glens Falls Hospital, Warren County, NY.

The patient described his right flank and lower back as having sharp and severe pain that came on in waves and lasted for 20-125 minutes before easing. Upon inquiry, he denied dysuria, hematuria, or history of kidney stones. He did not report fever or jaundice, and denied any recent trouble with his bowels, being nauseous, or having changes in bowel habits. With the exception of a previous appendectomy that was well-healed, he did not have any past history of medical problems (including HIV), nor was he on any medication, including over-the-counter non-steroidal anti-inflammatory drugs (NSAIDs). The patient reported an allergy to acetylsalicyclic acid (aspirin), which he said gave him a rash. The patient’s recent travel history was recorded, but animal contact was not reported until later (after his rabies diagnosis) by a family member. Review of his systems was negative from the gastrointestinal standpoint during these consultations, and his vital signs were stable (Table 1). Although he was being evaluated for kidney stones, other differential diagnoses listed at that point included musculoskeletal pain.

On September 29 (12 hours post-arrival to the hospital), the patient’s vital signs begin to reveal an autonomic instability (Fig. 1). A slight elevation of body temperature (Table 1) and signs of cardiac arrhythmia were noted. Approximately 2 hours later, he was diaphoretic with a slight fever and was unable to take in food or liquids (Fig. 1). He also reported significant discomfort and pain in his right flank/abdominal region. By 7:30 p.m. he became restless and agitated. He reported pain in his right hip/flank and throat irritation, although upon airway and mouth inspection, there was no redness or swelling. He was able to take a lorazepam oral tablet and tolerate thickened liquids (i.e., milkshake, ice cream) but not fluids.

When he was formally admitted (September 30, day 6) at 1 a.m., the patient became very restless, wandering around the hall stating that the “bed is too hot” and that he needed to “sleep outside.” Although he was told to climb back into bed, a few minutes later a nurse found him sleeping on the floor. He was found to be alert and oriented with stable vital signs. Although he denied any pain, he requested medication to “put him at ease.” Five hours later the patient reported that he was drowning and that he couldn’t breathe, although his oxygen saturation was normal (Table 1). His back and abdominal pain returned several hours later, at which point he appeared diaphoretic, had difficulty catching his breath and swallowing liquids, and was extremely anxious. In addition, he stated that his legs were “numbing up.” Elevations of all vital signs, including a fever, were noted (Table 1). The patient’s diaphoresis, confusion, and hallucination became progressively worse. Between 5 p.m. and midnight, he was unsteady on his feet and became psychotic, which warranted his transfer to the behavioral health unit (Fig. 1). His sweating (especially on his face and chest), shakes, and anxiousness became more constant, which he stated was normal for him. At one point he managed to eat a small portion of a sandwich with some difficulty swallowing but was completely unable to swallow liquids. Because of his constant need to get up from the bed he was placed under a one-on-one security observation.

The next morning October 1 (day 7) the patient became completely incoherent. He made many attempts to get out of bed, which required constant observation for safety. At one point he became combative and belligerent, striking staff members, and yelling incoherently in his native tongue (Fig. 1). Due to his combativeness, he was put on a four-part restraint and intravenous hydration was initiated. A physical examination revealed extreme diaphoresis on his face but he was dry on other parts of his body. His abdomen was distended, although bowel sounds were present. He was tachycardic with rigidity and tremor but without dystonia.

Four hours after being placed on restraints, he was alert and calm, but continued to have hallucinations. He was debriefed about removing the restraints and nodded in response. He remained calm after the restraint removal. An hour later, he appeared to be sleeping with his eyes rolled inward. His extremities were rigid and there was heavy frothing around the mouth. Cardiac arrest ensued but he was taken to the intensive care unit (ICU), successfully resuscitated, and placed on ventilatory support (Fig. 1). Blood chemistry values were abnormal, with elevation in glucose, urea, creatinine, sodium, and chloride (Table 2). The proportional increase in monocytes and decrease in lymphocytes continued. Red blood cells, hemoglobin, and hematocrit were below normal for the first time. Blood work and urinalysis taken from a catheter revealed an elevated sedimentation rate and glucose and protein levels with a moderate level of blood (Table 3).

On October 2 (day 8), the patient experienced multiple episodes of sinus arrhythmia with atrial ventricular (AV) block. Since his overnight increased temperature, his heart rate had been fluctuating between bradycardia and tachycardia. Although the patient was able to open his eyes, he was unable to follow any commands. With the increase in WBCs to 12.3 × 10^3^/uL (Table 2), a number of infectious diseases were under consideration, including primary pneumonia, rabies, HIV, and herpes simplex. A toxicology screen was not done. Information on any possible illicit drug use was not available. The patient had indications of a previous Herpes simplex virus infection (IgG titers for both HSV-1 and HSV-2 were 2.1 EIA units). With the patient unable to provide consent, HIV testing was not done, but a CD_4_ lymphocyte count was low normal (234 cells/mm^3^).

Medical treatment for the pneumonia included ceftriaxone. The suspicion of rabies was increased based on the hydrophobia, foaming at the mouth, and rapid downhill course. The hospital infection control officer put the patient on droplet precautions and initiated an evaluation of rabies infection. A corneal impression, skin biopsy from the nape of the neck, sputum sample, and saliva sample were taken for submission for rabies testing.

**Figure 2:**
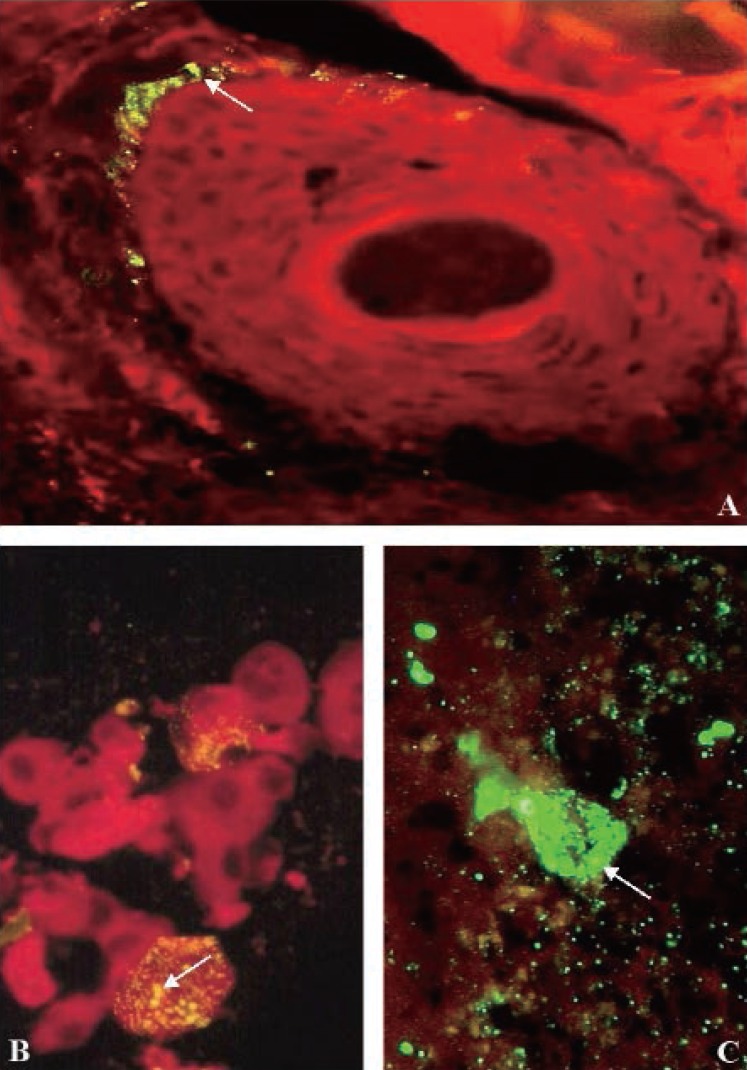
(A) Immunofluorescence evidence of rabies viral protein in antemortem skin biopsy sample. Inclusions of rabies virus antigen are disclosed in cytoplasm of sensory nerves surrounding hair follicle. Specific staining with FITC-labeled, rabies nucleocapsid antigen-specific monoclonal antibodies appears as characteristic apple-green fluorescence (arrow). With Evans blue counterstain, ×250 magnification; (B) Immunofluorescence evidence of rabies viral protein in antemortem corneal impression. Specific staining with FITC-labeled, rabies antigen-specific monoclonal antibodies appears as yellow-green fluorescence of inclusions in cytoplasm of corneal epithelial cells (arrow). With Evans blue counterstain, ×250 original magnification; (C) Immunofluorescence evidence of rabies viral protein in frozen section of unfixed postmortem cerebellum specimen. Specific staining with FITC-labeled rabies viral nucleocapsid protein-specific monoclonal antibodies, appearing as characteristic apple-green fluorescence of numerous intra-cytoplasmic inclusions in the large Purkinje cell body (arrow). With Evans blue counterstain, ×250 original magnification.

A second corneal impression and skin biopsy were again taken on October 3 (day 9). Nuchal skin biopsies and corneal impressions provided a positive antemortem diagnosis of rabies on October 3 (Fig. 2A, 2B). The laboratory results were reported to the attending physician as positive on October 3 (Fig. 1) and the issue of a DNR classification and discontinuation of ventilation were discussed with family members. Acyclovir was begun IV (10 mg/kg body weight every 8 hours), which was subsequently discontinued on October 5.

The patient was heavily sedated and appeared stable with ventilatory support. He was still unable to follow any verbal commands despite being able to slightly open his eyes. By October 4 (day 10), he appeared unresponsive although he had mid-pupillary response to tactile stimuli and he squinted when his corneas were touched. A physical examination revealed clear lungs, a soft abdomen, and a decreasing blood pressure (Table 1). By October 7 (day 13), the patient was not responding at all, had no noticeable reflexes, signs of hypothermia and bradycardia (Fig. 1) On October 8, family members had arrived at the hospital and a DNR order was formally issued. On October 9 (day 15), patient’s heart rate slowed to asystole, and the patient was pronounced dead by the attending physician.

Subsequent molecular characterization using the saliva sample revealed rabies viral RNA bearing a close genetic similarity to the African dog variant (8). Postmortem examination was performed but not required in this case. Postmortem examination is of particular value if antemortem testing is tenuous or when initial diagnosis is based solely upon evidence of antibody in serum and CSF, which does not permit antigenic or genetic identification of the responsible Lyssavirus genotype or rabies virus variant (2).

**Figure 3:**
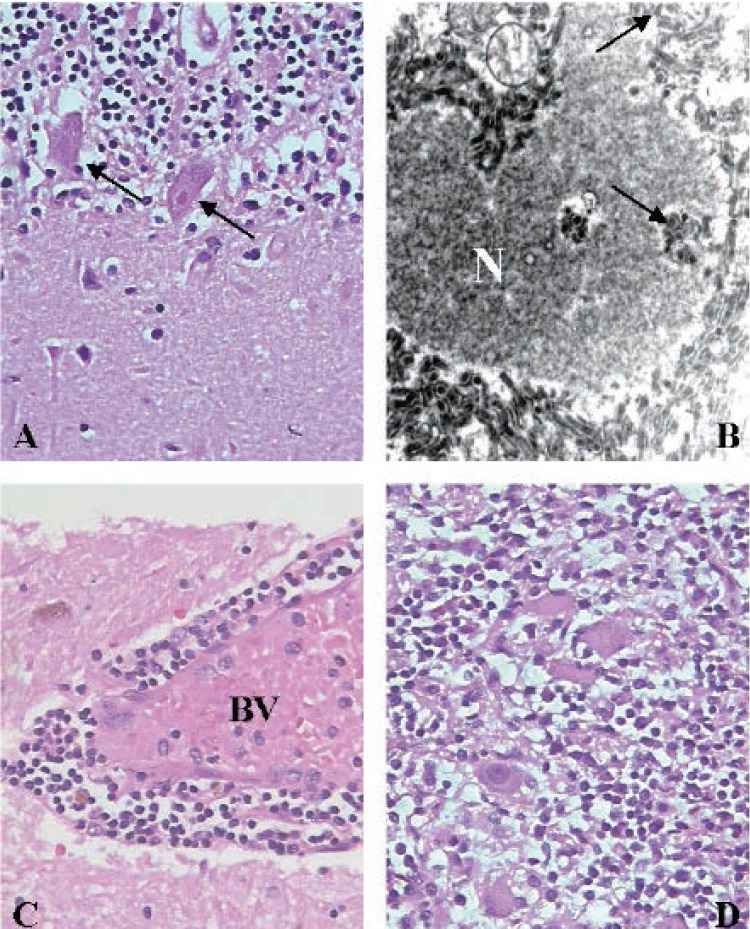
Photomicrographs of rabies infection involving the nervous system. (A) Cerebellar Purkinje cells infected by rabies virus containing intracytoplasmic eosinophilic Negri bodies (arrows). (B) Electron micrograph showing characteristic bullet-shaped intranuclear and cytoplasmic viral particles (arrows) in cross and longitudinal profiles (N = nucleus). (C) Perivascular lymphocytic cuffing in substantia nigra (BV = lumen of blood vessel). (D) Lymphohistiocytic ganglionitis in the paravertebral sympathetic ganglion. Original magnifications: ×400 (A, C, D), and ×40,000 (B).

Examination of the nervous system tissue taken at autopsy demonstrated an acute encephalomyelitis and confirmed the antemortem diagnosis of rabies (Fig. 3). Diagnostic Negri bodies were identified in neurons throughout the central nervous system, most prominent in the Purkinje cells of the cerebellum (Fig. 3A). These intracytoplasmic inclusions were composed of rabies viral proteins as confirmed by immunofluorescence antibody testing (Fig. 2C). Ultrastructurally, the infected neurons contained viral particles in both cytoplasmic and nuclear compartments (Fig. 3B). In addition, there was wide-spread perivascular cuffing with lymphohistiocytic infiltrates in the brainstem and spinal cord (Fig. 3C). Lymphohistiocytic infiltrates were also found in the autonomic nerve plexus of the epicardium and in the paravertebral sympathetic chain ganglia (Fig. 3D).

To determine the patient’s exposure to rabies, NYSDOH attempted to locate contacts of the patient in Ghana. Family members who were on their way to the hospital provided the name of a contact person in Ghana to the patient’s physician. NYSDOH was able to reach this person in Ghana by telephone on October 6. He reported that the patient, the patient’s nephew, and two university students were all bitten by a puppy in June which subsequently died (additional puppies had also died of an unknown cause in the same weeks). In the subsequent days, NYSDOH was able to reach the Ghana Ministry of Health and officials at the patient’s home university in Ghana, who were able to locate the nephew. He had not yet developed any signs of rabies from being bitten by the same dog as the patient, but was given prophylactic treatment beginning on October 12. The university students could not be located and they were lost to follow-up.

### Human-to-Human Transmission Control

Upon the diagnosis of rabies, public health officials and hospital administrators initiated a mass interview of people for possible exposures and postexposure prophylaxis (PEP). In most cases, PEP consists of five doses of rabies vaccine over the period of a month and one dose of rabies immune globulin at the beginning of treatment (9). There may be temporary shortages of PEP biologics. PEP is also expensive and may have adverse reactions. Thus, PEP is recommended only for specific known rabies exposure routes (9). The hospital reported widespread fears among hospital staff, ranging from medical to food service, clerical, and janitorial personnel. Many of the staff members spent considerable time reviewing and consulting with hospital infection control about the details of all their activities related to the patient since he had been hospitalized. The psychological impact of the diagnosis also extended to family members of hospital staff. For instance, one hospital staff member involved in case-patient transport was told by his family that he could not return home until he received rabies treatment.

The fear surrounding postmortem handling was especially strong. Despite information and reassurances from local and state health staff and hospital infection control personnel about the minimal risks and appropriate precautions to eliminate them, many local and regional pathologists, ambulance companies, and funeral homes refused acceptance of the body. One local health commissioner denied transport of the body into the county for funeral service upon being notified that the patient died of a communicable disease. These arrangements required considerable public health time, consultation, and education.

In addition, the patient’s widespread travel needed to be assessed. Through an interview with the decedent’s friend who met him when he got off a bus in Buffalo, it did not appear that the patient was aggressive when he was on the overseas plane or bus. Although he appeared anxious and agitated, which was interpreted by family and friends to be related to personal stressors, the patient appeared normal. In addition, there had been no reports of aggressive passengers on either the plane or the bus. Thus, it was believed unlikely that the types of contacts of highest concern, particularly biting, occurred in either public setting. The patient was described by his friend as a formal person who would not normally have physical contact with other people on a plane or bus, such as hugging, kissing, sharing drinking glasses, etc. Thus, the interview process coordinated by hospital staff with local and state health staff focused primarily on approximately 120 hospital contacts.

To assess exposure, the local health, state health, and hospital team developed a rabies PEP fact sheet and questionnaire that focused on whether the healthcare workers had been bitten or kissed by the patient, whether they were in contact with the patient’s fluids or secretions, and specific procedures performed on the patient. Using the fact sheet and questionnaire, 25 individuals from the hospital plus the decedent’s friend were ultimately given PEP treatment beginning on October 4. Half of these persons were recommended for treatment due to their direct contact with the patient’s saliva or mucous membranes. The other half requested PEP due to their concerns about possible aerosol transmission of rabies virus while in close proximity to the patient during certain medical procedures, including the resuscitation efforts. No cases of rabies aerosol transmission between humans have ever been reported; however, two cases of rabies were reported from Ethiopia, one in a mother subsequent to caring for her child dying of rabies (10). The other case was of a 5 year old who died of a clinical syndrome consistent with rabies infection following repeated kisses from his mother who died from rabies after being bitten by a rabid dog (10). Aerosol transmission was one of the potential transmission routes in laboratory-acquired human infections (11) and two human cases from a bat cave exposure (12, 13). Mortuary staff and pathologists did not require PEP because they used gloves, masks, and eye protection when handling the patient.

None of these 26 vaccinated individuals contracted rabies. There were reports of mild to moderate reactions from four of those receiving PEP after the second and third vaccinations. These reactions included headache, body ache, joint stiffness, fatigue, cough, head congestion, vomiting, and diarrhea.

## DISCUSSION

This case was compared with 46 other U.S. rabies cases (14-45) on four factors: the duration between onset and death, chief complaints, other diagnoses, and stage of rabies diagnosis, and the detailed information on these factors is provided for the 20 rabies deaths that were diagnosed antemortem similar to the NY case (Table 4). The NY case had a symptom duration (between onset and death) of 15 days, compared to a mean of 16.2 days in other cases with a range of 6–43 days. As indicated in other cases, initial complaints are usually nonspecific and may include fever, nausea, vomiting, dyspnea, cough, chills, myalgias, sore throat, hand/arm weakness and numbness, and headache. Focal abdominal pain has been reported (14). Signs may also include generalized itching, a gagging sensation, speech stuttering, and episodes of staring and unresponsiveness lasting 10-15 seconds. Pain and paresthesias in the area of exposure are often noted, as in the NY case with pain being predominantly right-sided (bite wounds were on the right leg and arm). Classic signs of rabies, such as hydrophobia, hallucination, anxiety, agitation, confusion, and increased body temperature (36°C-39°C), are not immediately noticeable until after approximately four days post-symptom onset (15-37). Many of the initial diagnoses include viral upper respiratory illness, panic disorder, drug overdose, tetanus, musculoskeletal pain, bilateral ear effusions, cerebrovascular accident, unspecified anxiety disorder, and encephalitis (Table 4). Rabies is considered in the differential diagnosis for only a very small number of cases. Of the 48 cases since 1989, more than half (26) were diagnosed postmortem, including the recent five cases associated with organ transplantation (38, 39). The cases diagnosed antemortem were generally diagnosed very late in the clinical period, usually 2-3 days before death.

**Table 4:** U.S. Human Rabies Deaths, Antemortem Diagnosis, 1991-2003

Cases (reference)	Clinical Duration (Days)	Chief Complaints	Differential Diagnoses	PEPs

TX, 1991 (24)	14	Shortness of breath, difficulty swallowing	Panic disorder; rabies	43
CA, 1992 (37)	18	Shoulder pain from injury	Rabies	17
CA, 1993 (36)	15	Pain in left jaw (spider bite?), chest, and shoulder; sore throat, insomnia, nausea, vomiting, can’t eat/drink	Chest pain, anxiety disorder-unspecified	33
WV, 1994 (25)	13	1-day history of shaking, speech difficulties, unable to drink, vomiting, severe anxiety, muscle tremors (made 2 visits to hospital)	Tetanus, viral encephalitis, acute hemorrhagic encephalitis, drug toxicities or withdrawal	48
FL, 1994 (18)	141 (appx)	Severe neck pain and headache, epigastric pain, chest & back pain	Acute renal failure from mesangial proliferative glomerular nephritis; meningitis; CNS vasculitis	16
TN, 1994 (15)	16	Influenza-like symptoms, recurring back pain, left-sided chest pain, left arm paresthesia, chest & breast numbness, shaking, abdominal cramps, headache, lower back pain (5 visits to hospital)	Herpes zoster; bronchitis with pleurisy; anxiety & lower back strain; aseptic meningitis; rabies 2 days before death	47
WA, 1995 (30)	10	2-day history of drowsiness, listlessness, abdominal pain, anorexia, sore throat, pain on left side of neck (made 2 visits to hospital)	Rhinitis, bilateral conjunctivitis; dehydration; drug intoxication; sepsis; viral encephalitis; rabies 2 days before death	72
CT, 1995 (29)	16	General fatigue, stiffness, tremors, tingling in left arm & hand; low-grade fever, neck pain, pain left side of face (2 visits to hospital)	Cervical radiculopathy; Lyme meningoencephalitis with peripheral nerve involvement; rabies 8 days before death	83
CA, 1995 (19)	13	1-day history of vomiting & severe headache, sore throat (made 4 visits to hospital)	Cephalgia; nonspecific encephalitis; rabies 9 days before death	12
FL, 1996 (20)	42	Anxiety, difficulty breathing while speaking, left lower-quadrant abdominal pain, left leg pain, lower back pain, and lethargy (2 visits to hospital)	Constipation; rabies	4
MT, 1996 (34)	16	Fever, sore throat, productive cough, severe right-sided supraorbital pressure & tenderness for several weeks (2 visits to hospital)	Sinusitis; pneumonia; severe hyponatremia; presumptively viral encephalitis	26
NH, 1996 (26)	11	2 days paresthesias and pain radiating up left arm from the site of a healed dog bite in Nepal,difficulty breathing, throat spasms, nausea, vomiting (2 visits to hospital)	Left cervical radiculopathy	7
NJ, 1997 (31)	12	Aching sensation in right shoulder & neck, vomiting, chills, sore throat, fever, insomnia, dysphagia (made 3 visits to hospital)	Febrile syndrome; tetanus; herpes encephalitis; rabies 7 days before death	50
VA, 1998 (33)	18	Malaise & back pain while working on a roadside cleanup crew; muscle pains, vomiting, abdominal cramps (made 2 visits to hospital)	Intoxication with anticholinergic agents such as pesticides or Jimson weed;rabies 11 days before death	48
CA, 2000 (35)	6	2-days of increasing right arm pain & paresthesias (2 visits to hospital)	Atypical neuropathy; rabies 4 days before death	NA
MN, 2000 (35)	12	6 days worsening right arm pain and paresthesias	Nerve conduction studies were consistent with carpal tunnel syndrome	NA
CA, 2002 (23)	14	Headache, jaw pain, photophobia, dizziness, numbness, nausea, vomiting, agitation (made 2 visits to hospital)	Dehydration; rabies 5 days before death	46
IA, 2002 (21)	13	Nausea, vomiting, generalized abdominal pain, shortness of breath, headache, back stiffness (2 visits to hospital)	Anxiety; suspected drug reactions or withdrawal syndrome	124
TN, 2002 (17)	11	Headache, neck pain, right arm numbness & weakness; slight fever (2 visits to hospital)	“Muscle strain”; rabies 5 days before death	23
CA, 2003 (41)	20 (appx)	Atypical chest pain; 2 weeks mild, nonspecific complaints, 5 days right arm pain & paresthesias, 1 day right-hand weakness	Rabies	44

NA, information not available; PEPs, number of human rabies prophylaxes (PEPs) related to contact with human case.

Since 1980 the average number of PEP treatments for human rabies cases has been 64.6 (20). Of the 48 cases since 1989, 28 had a lower than average number of PEPs per case. For the NY case, the use of a defined questionnaire to interpret exposures may have been effective in reducing PEP requests to 26. PEPs appear to be lower with early suspicion of rabies, use of a predefined questionnaire, patients’ limited social contacts, and prompt initiation and maintenance of protective barrier techniques during presentation and hospitalization. In most of the cases, health care workers made up the largest treatment group, primarily due to the rapid clinical progression that resulted in hospitalization and intense supportive therapy.

The Centers for Disease Control and Prevention (CDC) estimated that the public health costs for rabies in the U.S. exceeds $300 million annually (46), primarily due to domestic animal vaccinations, wild animal control programs, rabies laboratory testing, and medical care such as PEP. For a single human PEP, the national cost estimate ranges from $1,039 to $4,447 per year (47). In NYS, the average cost per person for PEP increased from $769 in the 1993 fiscal year to $1136 in 1998 (48). During the six-year period, it was estimated that approximately $13.9 million was spent in NYS to prevent rabies, of which 77% ($10.7 million) was spent for PEP. Using the 1998 average estimate of $1136 per PEP for the costs of purchasing biologics, the PEP expenses associated with treating contacts of the NY 2000 case approximates $30,000, excluding expenses associated with the administration of the treatment, time for consultation and evaluation of exposures, time lost for the usual five treatment medical visits, transportation, lost wages, and the patient’s medical bill.

The need to clearly identify individuals for prophylaxis is critical not only for preventing any theoretical possibility of human-to-human transmission, but also to appropriately control the health care costs as well as the psychological fears associated with human rabies cases. Thus, early diagnosis of rabies and the implementation of timely interviews and appropriate questionnaires for human contacts are important. Case definitions of exposure must be clearly identified. In this case, the case definition of exposure was “bite, scratch, or direct contact between the patient’s saliva and an open wound or mucous membrane.”

To prevent rabies, exposure to wild animals should be avoided, domestic animals should be current on vaccinations, and if a person is exposed to rabies, proper wound care and prompt rabies treatment is critical. These core principals of rabies prevention were breached in the NY case. In many of the U.S. cases there were delays of up to 2-12 days after symptom onset in seeking medical attention (15, 16, 18, 24, 27, 28, 36, 37). Consequently, early diagnosis is limited as indicated by many of the cases having rabies considered in the differential diagnoses very late in their clinical progression and/or only upon postmortem laboratory confirmation. In such situations, it can be difficult to evaluate human contacts and exposures to the case, and difficult to limit the number of those exposed through contact precautions. Thus, in any case of acute, rapidly progressing encephalitis, which may develop 4-5 days after the onset of a non-specific prodrome, rabies must be included in the differential diagnoses, even if the patient does not recall being bitten by an animal. If rabies exposure is suspected, urgent administration of rabies immune globulin and vaccination are needed, provided the clinical signs of rabies are not present (9). Also, the local health authority or state public health laboratory should be contacted to arrange for antemortem or postmortem laboratory testing which may include examination of samples of nuchal skin biopsy, corneal impressions, saliva, serum, CSF or brain tissue.

A final reason to improve early consideration and diagnosis of human rabies cases is the possibility of treatment to reduce the case fatality rate. There has been one recent case reported of a 15-year old girl who survived bat rabies after induction of coma and treatment with ketamine, midazolam, ribavirin, and amantadine (40, 44). However, the bite wound was recognized by the patient and family at the time of the bite, and it was washed with peroxide. Thus, the effectiveness of the medical treatment versus other factors including the patient’s early and strong antibody response cannot be determined in this case. Rabies virus was also never isolated from the patient. Repeated applications of this type of medical treatment will need to occur to draw conclusions about its effectiveness.
